# VIRUSBreakend: Viral Integration Recognition Using Single Breakends

**DOI:** 10.1093/bioinformatics/btab343

**Published:** 2021-05-11

**Authors:** Daniel L Cameron, Nina Jacobs, Paul Roepman, Peter Priestley, Edwin Cuppen, Anthony T Papenfuss

**Affiliations:** Bioinformatics Division, Walter and Eliza Hall Institute of Medical Research, Parkville, VIC 3052, Australia; Department of Medical Biology, University of Melbourne, Melbourne, VIC 3010, Australia; Hartwig Medical Foundation Australia, Sydney, NSW 2060, Australia; Hartwig Medical Foundation, Amsterdam 1098, The Netherlands; Hartwig Medical Foundation, Amsterdam 1098, The Netherlands; Hartwig Medical Foundation Australia, Sydney, NSW 2060, Australia; Hartwig Medical Foundation, Amsterdam 1098, The Netherlands; Center for Molecular Medicine and Oncode Institute, University Medical Center Utrecht, Utrecht 3584, The Netherlands; Bioinformatics Division, Walter and Eliza Hall Institute of Medical Research, Parkville, VIC 3052, Australia; Department of Medical Biology, University of Melbourne, Melbourne, VIC 3010, Australia; Peter MacCallum Cancer Centre, Melbourne, VIC 3000, Australia; Sir Peter MacCallum Department of Oncology, University of Melbourne, Melbourne, VIC 3010, Australia

## Abstract

**Motivation:**

Integration of viruses into infected host cell DNA can cause DNA damage and disrupt genes. Recent cost reductions and growth of whole genome sequencing has produced a wealth of data in which viral presence and integration detection is possible. While key research and clinically relevant insights can be uncovered, existing software has not achieved widespread adoption, limited in part due to high computational costs, the inability to detect a wide range of viruses, as well as precision and sensitivity.

**Results:**

Here, we describe VIRUSBreakend, a high-speed tool that identifies viral DNA presence and genomic integration. It utilizes single breakends, breakpoints in which only one side can be unambiguously placed, in a novel virus-centric variant calling and assembly approach to identify viral integrations with high sensitivity and a near-zero false discovery rate. VIRUSBreakend detects viral integrations anywhere in the host genome including regions such as centromeres and telomeres unable to be called by existing tools. Applying VIRUSBreakend to a large metastatic cancer cohort, we demonstrate that it can reliably detect clinically relevant viral presence and integration including HPV, HBV, MCPyV, EBV and HHV-8.

**Availability and implementation:**

VIRUSBreakend is part of the Genomic Rearrangement IDentification Software Suite (GRIDSS). It is available under a GPLv3 license from https://github.com/PapenfussLab/VIRUSBreakend.

**Supplementary information:**

Supplementary data are available at *Bioinformatics* online.

## 1 Introduction

As made abundantly clear by the SARS-CoV-2 and HIV pandemics, viral infections constitute a major worldwide threat to human health. While most viruses do not integrate into the host genome, there is a significant global health burden caused by the subset of those that do, especially in cancer (McLaughlin-Drubin and Munger, 2008). For example, human papillomavirus (HPV) is present in the majority of cervical cancers, Merkel cell polyomavirus (MCPyV) is the primary cause of Merkel cell carcinoma, and the Epstein-Barr virus (EBV) infects around 90% of the human population and is associated with multiple forms of cancer (Khoury *et al.*, 2013). Other oncoviruses include Kaposi’s Sarcoma-associated herpesvirus (HHV-8), and Hepatitis B virus (HBV)—the leading cause of Hepatocellular carcinoma (HCC). For some of these, the specific location of the viral integration is a direct driver of oncogenesis and influences tumour phenotypes. For example, HBV integrations in the TERT promoter region are associated with high telomerase expression and cancer cell survival (Zapatka *et al.*, 2020). This integration site-specific behaviour is not just limited to oncogenic viruses, as human immunodeficiency virus (HIV) elite controllers have shown to have a high rate of centromeric viral integrations (Jiang *et al.*, 2020). The reliable detection of viral integrations anywhere in the genome is key to understanding the effect of viral integration to disease.

Recent advances in sequencing technology have made routine large-scale whole genome sequencing (WGS) possible, including tumour sequencing (Priestley *et al.*, 2019). These WGS datasets enable the detection of viral integrations through the identification of structural variant breakpoints between the host genome and the viral sequence. While there exist several tools capable of detecting viral integrations in WGS data, these tools have not yet gained widespread adoption. Existing tools fall short in one or more of three areas: the ability to detect more than one virus or virus family, runtime performance and the inability to detect integrations into repetitive regions of the host genome (such as centromeres).

At a high level, WGS viral integration detection software finds integration sites by identifying clusters of reads or read pairs spanning from host reference sequence to viral reference sequence. Viral integration tools such as BatVI (Tennakoon and Sung, 2017), VirTect (Xia *et al*., 2019) and Virus-Clip (Ho *et al*., 2015) require a viral reference as input. While some of these tools are true single-virus tools, others can in theory be configured with multiple viral reference genomes. Including related viruses causes read alignment ambiguities when these viruses contain homologous regions. VirusFinder (Wang *et al*., 2013), VirusFinder2/VERSE (Wang *et al*., 2015) and VirusSeq (Chen *et al*., 2013) avoid this problem by first identifying viral presence before proceeding to integration detection using a single viral reference genome. These tools are still limited to a single viral reference genome, so HHV-6 infection may mask the presence of a short genome such as HBV. The Pan-Cancer Analysis of Whole Genomes (PCAWG) project (Zapatka *et al*., 2020) avoided this problem by performing viral read classification prior to viral integration detection but this pipeline was not publicly released as a standalone tool and its integration detection performance is determined by their use of VERSE as the sole integration detection tool. The need for an integrated, easy to use, virome-wide integration detection was identified by Chen *et al*. (2019), a gap we propose to fill with VIRUSBreakend.

For the vast majority of whole genome sequencing projects and emerging diagnostics, viral integration detection is only one part of a larger analysis. Tools that are not computationally efficient will struggle to gain widespread adoption. Tools such as BatVI and Virus-Clip were developed in direct response to the computational cost of tools such as VirusFinder, VERSE and VirusSeq. By far, the most computationally expensive step is the alignment of reads to the host and viral genomes and multiple approaches have been taken. VERSE, VirusSeq and ViralFusionSeq (Li *et al*., 2013) use a host then virus alignment approach, BATVI and Virus-Clip use a virus then host approach, while ViFi (Nguyen *et al*., 2018) and VirTect (Xia *et al.*, 2019) take a combined host and virus approach. Each of these approaches have their advantages and drawbacks, but host then virus alignment approach has the unique advantage that it uses as input a bam file that will almost certainly have been generated in a typical WGS pipeline. Here, we show that this approach can reduce the real-world computational cost of incorporating viral integration detection to less than one tenth of the computational cost of realignment.

**Fig. 1.**
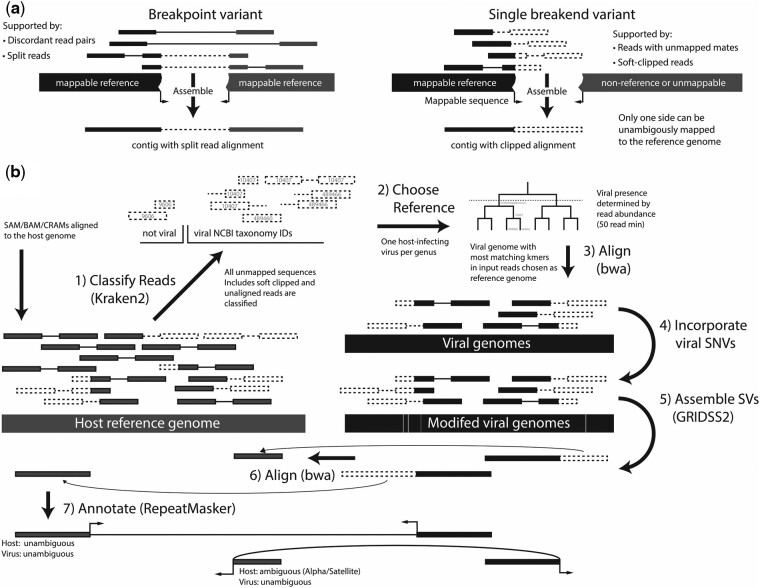
VIRUSBreakend Overview. (**a**) Comparison of breakpoint and single breakend variant calling. Single breakends are breakpoints in which only one side can be unambiguously placed. (**b**) VIRUSBreakend workflow. (1) Sequences not aligned to the host are taxonomically classified to identify viral abundance. (2) The most abundant host-infecting viral taxon per genus is identified and a viral reference genome chosen based on greatest kmer-based similarity to the viral reads. (3) The full read pairs for all viral sequences are aligned. (4) SNVs are called and incorporated into the viral reference. (5) Single breakends variants are assembled and called. (6) Host integration sites identified by alignment of the breakend assemblies. (7) Ambiguous/multi-mapping host integration sites are repeat annotated. This virus-centric approach allows identification of integrations in repetitive/low mappability host regions

Finally, and most crucially, all existing viral integration detection tools rely on clusters of host-aligned reads. Even tools such as VirusFinder/VERSE that perform an assembly step, still relying on read host alignment clusters to determine the insertion site. This fundamentally limits the viral integration detection capability in host regions with low mappability. Ignoring reads derived from regions that have low mappability will result in false negatives. Reporting either a single arbitrary alignment or all possible alignments of a multi-mapping reads with both result in a high false positive rate and overestimation of the number of insertion sites. Our solution to this problem is to utilize single breakend variant calling on the viral reference genome. The Variant Call Format Specification (VCF) (Danecek *et al.*, 2011) file format defines a breakend as a structural variant with a genomic position and orientation. A breakpoint consists of a pair of breakends, whereas a single breakend variant consists of only one breakend with the other side unable to be unambiguously placed with respect to the reference (Fig. 1a). This can be due to multiple possible reference locations, or a breakpoint to non-reference sequence. We utilize the latter. By first identifying where in the viral genome an integration site occurs and assembling the host sequence adjacent to the integration, we obviate the problem of multi-mapping host read alignments. In cases where the host location cannot be unambiguously determined from the assembled contig, the contig sequence provides information about the repeat context of the integration site.

Here we present a novel single breakend-based approach that can reliably detect viral presence and integrations anywhere in the host genome. By identifying and assembling single breakend variants in the virus genome followed by taxonomic classification and alignment of the breakend contigs, VIRUSBreakend is able to reliably identify viral integrations in regions inaccessible to current integration detection approaches.

## 2 Materials and methods

VIRUSBreakend uses a multistage approach to identifying viral insertions (Fig. 1b). Starting with a host-aligned SAM/BAM/CRAM file, VIRUSBreakend identifies viral reads of interest through Kraken2 (Wood *et al*., 2019) taxonomic classification of all unaligned or partially aligned sequences using a custom Kraken2 database. If the read is at least partially classified as a virus, the full read pair is considered for further analysis. This approach allows read pairs in which either read contains any viral sequence to be efficiently identified. The custom Kraken2 database consists of the default human, bacteria, viral and UniVec_Core databases as well as all NCBI viral neighbour genome assemblies (https://www.ncbi.nlm.nih.gov/genome/viruses/about/assemblies/) (Brister *et al.*, 2015). The inclusion of viral neighbour genomes is essential for HPV subtype resolution as some subtypes (e.g. HPV-45) do not have a RefSeq assembly.

A viral reference fasta is then created that includes a viral genome for each host-infecting genus containing a taxon to which Kraken2 assigned at least 50 reads. Viral reads are compared to all viral reference genomes associated with the identified taxon or its descendents and the genome with the most matching viral read kmers is chosen as the viral reference genome for that genus.

SNVs called with bcftools and the viral reference modified to incorporate these SNVs. Viral read pairs are realigned (Li and Durbin, 2010) to the updated reference and structural variants called using GRIDSS2 (Cameron *et al*., 2017, 2020) and filtered to single breakends. In this context, single breakends are breakpoints in which one side cannot be unambiguously aligned to the viral reference genome. In VIRUSBreakend, these occur because the viral reference does not include the host genome. As well as the location and orientation of the break junction, GRIDSS2 reports the assembled sequence for every single breakend.

The assembled single breakend sequence is used to identify the host integration site by aligning to the host reference. Identified breakpoints fall into two categories: sites in which the host mapping is unambiguous, and ambiguous sites in which the integration site cannot be unambiguously determined (such as integrations into alpha satellite repeats). To facilitate downstream analysis, integration sites are annotated with the RepeatMasker (Smit *et al*., 1996) repeat type and class of the single breakend sequence.

The key advantage of this single breakend approach is that the initial variant calling is done independently of the integration site in the host genome. Only after the viral side of the integration site has been identified is the host integration location determined and this is determined through placement of the assembled contig not per read. This allows robust integration detection, including in repetitive regions of the host.

A detailed description of the methods is available in Supplementary Materials.

## 3 Results

### 3.1 Synthetic benchmark

To evaluate performance on idealised data, we created a synthetic benchmark with realistic integration sites. Viral integration sites were simulated by generating a fasta consisting of the 50kbp of host sequence before the insertion side, 2000 bp of HBV viral sequence, a 10 bp host gap, then the 50kbp of host sequence after the insertion site. To enable accurate simulation of integration in telomeric and centromeric repeats, chromosome 1 of the CHM13 Telomere-to-Telomere consortium assembly (Miga *et al.*, 2020) was used as the host sequence. The non-reference strain LC500247.1 was used as the viral sequence. 248 integration sites were simulated, one at each 1 Mb position along CHM13 chr1, each with a different HBV integration position. Simulated reads were generated using ART 2.5.8 (Huang *et al.*, 2012) to 5, 10, 15, 30 and 60 sequencing depth for a total of 1240 datasets. Each was called with VIRUSBreakend, VERSE, ViFi, BATVI and GRIDSS2 using their default hg19 settings. GRIDSS2 was included due to its favourable performance as a general-purpose breakpoint caller (Cameron *et al.*, 2019; Kosugi *et al.*, 2019) as well as to evaluate the performance of host-centric single breakend calling. True positives required host coordinates to match within 1000 bp (or 1 Mbp for locations without a 1-to-1 CHM13 to hg19 coordinate mapping) and viral coordinates to within 750 bp (to account for different HBV viral references). To ensure VIRUSBreakend was not favoured by its inclusion of LC500247 in its viral database, its viral database was restricted to RefSeq sequences only.

**Fig. 2.**
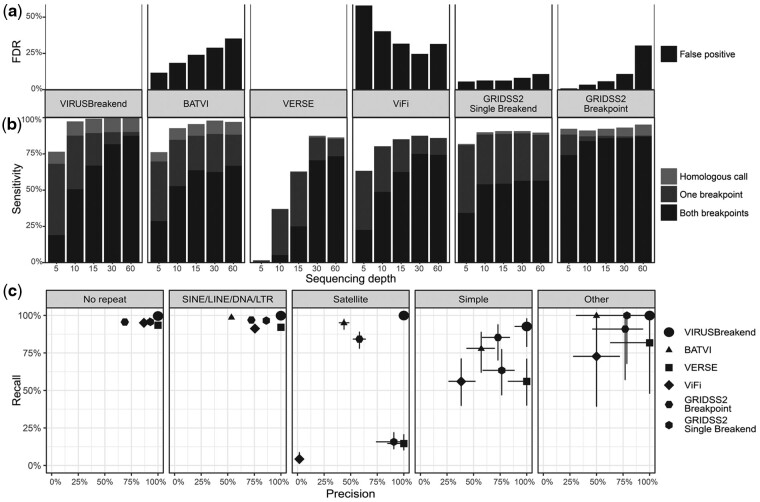
Simulation results. (**a**) Performance of simulated HBV integrations. 248 integration sites between the complete telomere-to-telomere CHM13 genome and HBV were independently simulated. Callers were run against hg19. Calls were considered homologous if the viral position matched but the integration site was at a homologous location in the human reference. GRIDSS2 single breakend calls are against a host-only reference, and GRIDSS2 breakpoint calls are against a combined host and viral reference. On this dataset, VIRUSBreakend and VERSE achieve perfect precision. (**b**) VIRUSBreakend has the highest overall sensitivity except at low (5×) sequencing depth. (**c**) Caller precision/recall at 60× based on 2483 simulated integration sites. VIRUSBreakend’s single breakend approach achieves high precision and recall across all repeat classes, including satellite repeats

ViFi and VIRUSBreakend have negligible false positive rates in this simulation (Fig. 2a), while BATVI and GRIDSS2 show similar false discovery rate (FDR) trends that increase with sequencing depth. ViFi shows the opposite with a higher FDR rate at low depth (Fig. 2a). With each insertion event having two breakpoints, true positives were broken down into the detection of both sides, only one side and the detection of the insertion but at a homologous location in the reference genome with homologous calls defined matching only viral coordinates. In this simulation, VIRUSBreakend achieved an overall recall above 99% by 15× sequencing depth with the next closest, BATVI reaching 98% at 30× depth (Fig. 2b). VIRUSBreakend, BATVI, VERSE and ViFi show similar recall trends with sensitivity increasing with sequencing depth, with GRIDSS2 recall relatively unaffected by depth. Except for high sequencing depth VIRUSBreakend and GRIDSS2 breakpoint calls, callers could not reliably detect both sides of the integration sites. Downstream analysis pipelines may have to recover low quality/filtered calls near known integration sites to fully reconstruct complex integrations.

**Fig. 3.**
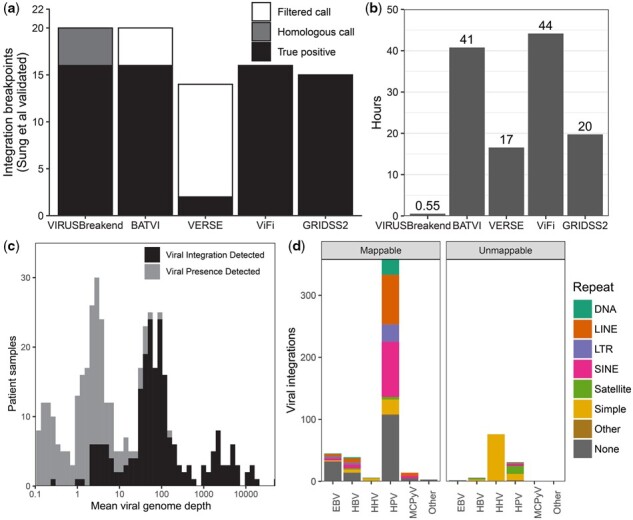
WGS results and runtime performance. (**a**) Sensitivity on the 22 integration sites validated by Sung *et al.* (2012) in a hepatocellular carcinoma cohort. The two false negatives were the two sides of a viral integration that VIRUSBreakend disagreed with the Sung *et al.* host position. (**b**) Runtime on sample 177 T from the hepatocellular carcinoma cohort when allocated 4 cores and 64 GB memory. The cost of the initial host genome alignment is not included for VIRUSBreakend or VERSE as this is typically performed in a WGS pipeline regardless of whether viral integration detection is performed or not. (**c**) Distribution of viral genome depth of coverage across 5191 metastatic tumour samples from the Hartwig Medical Foundation pan-cancer cohort. Viral integration sites were not found for samples with low viral depth of coverage. (**d**) Mappability of integration sites found in the Hartwig cohort. Unmappable sites have multiple candidate integration sites and are dominated by HHV integration into telomeric repeats and HPV integration

To determine the effect of the sequence context of the host integration, we simulated insertions at every 100Kbp along CHM13 chr1 (2483 integration sites) and evaluated callers at 60× sequencing depth. Breaking down caller performance by host integration site repeat annotation (no repeat, SINE/LINE/DNA/LTR, Satellite, Simple, Other) demonstrates that only VIRUSBreakend can reliably detect integration sites in simple and satellite repeats (Fig. 3c). Across all repeat types, VIRUSBreakend achieves perfect precision and higher sensitivity than the other callers and it is only in simple repeats that VIRUSBreakend sensitivity drops below 99%. In non-repeat sequence, VIRUSBreakend detect both breakpoints for 97% of integration sites, compared to 66%, 62%, 94%, 88%, 82% for BATVI, GRIDSS2 (single breakend), GRIDSS2, ViFi and VERSE. In abundant SINE/LINE/DNA/LTR repeats, VIRUSBreakend controls its FDR, identifying 1116 of 1117 integrations with no false positives whereas the other callers above 95% precision (BATVI 99%, GRIDSS2 97%, GRIDSS2 (single breakends) 97%) do so with a false discovery rate of 47%, 28% and 14% respectively (Supplementary Table S1). The trade-off between precision and FDR is even more evident in Satellite repeats in which VIRUSBreakend detects all 184 integration sites with no false positives whereas the other callers have high sensitivity, low FDR but not both (BATVI 95% sensitivity/56% FDR; GRIDSS2 84%/42%; GRIDSS2 (single breakend) 16%/9%; VERSE 14%/0%; ViFi 4%/98%).

### 3.2 Hepatocellular carcinoma benchmark

Next, we evaluated sensitivity on the 22 PCR/Sanger validated hepatitis B virus (HBV) integration site breakpoints from a hepatocellular carcinoma cohort (Sung *et al*., 2012, ERP001196) used by VERSE and ViFi. VIRUSBreakend identified 16 integration breakpoints, BATVI 16, ViFi 16, GRIDSS2 15 and VERSE 2 (Fig. 3a, Supplementary Table S2). Four of the validated integrations were entirely within HSATII or Beta satellite repeats for which VIRUSBreakend reported integrations into HSATII/Beta satellite repeats with a higher sequence similarity to the assembled viral integration sequence than the nominal integration site. Since the host PCR primer sequences chosen are present at both at the validated sites and the sites called by VIRUSBreakend, it is unclear where the actual integrations occurred. The locations are homologous and integration in either location would result in successful PCR amplification. Treating these homologous sites as correct calls, the sensitivity of VIRUSBreakend rises to 20/22. If calls internally filtered by VERSE and BatVI were included, sensitivity for these callers increased to 20/22 and 14/22 respectively. The two VIRUSBreakend false negatives were the two sides of an insertion site in which VIRUSBreakend reported an integration with matching viral coordinates but at a different host position than reported by Sung *et al.*

### 3.3 Analysis of a metastatic solid tumour cohort

To evaluate performance on a large pan-cancer cohort, we ran VIRUSBreakend on 5,191 tumour samples from the Hartwig Medical Foundation metastatic tumour cohort (Priestley *et al.*, 2019, DR-005, hartwigmedicalfoundation.nl/en/applying-for-data). Hartwig tumour samples were fresh frozen samples sequenced to 100× WGS on HiSeq X or NovaSeq sequencers. We detected viral presence in 610 samples, and viral integration in 160 of these. The most prevalent viruses were EBV with viral integration detected in 27 of 278 samples with viral presence, and HHV-6 (33 samples with integration/144 samples with detected virus), HPV-16 (60/93), HHV-7 (3/25) and HPV-18 (21/21) and HHV-5 (0/19). The likelihood of integration detection was driven primarily by viral depth of coverage with at least one integration site found in 95% (186/198) of samples achieving 10× viral depth (Fig. 3c).

Of the 43 cervical cancer in the cohort, HPV was detected in 38 with integration sites found in 37 (Fig. 3d). Anal (11/20 samples), penile (6/11) oropharynx (4/10), cancers also enriched for HPV presence. As expected (Travé and Zanier, 2016), the 5 cervical cancers without detected HPV were the only cervical cancers with TP53 driver mutations. Of the nine samples with HBV detected, seven were in liver cancers (*n* = 19) (all with detected integrations), which is consistent with previous findings (Zapatka *et al.*, 2020). Recurrent HBV integration was found in TERT (4 samples), and likely driver integrations found in/upstream of FOXP2, WNT2, EML6, ZDHHC11 and CTSC. Merkel cell polyomavirus integration was detected in all six Merkel cell carcinomas. HHV-8 was detected in the single Kaposi's sarcoma sample in the cohort, although the integration site was not.

Whether EBV is a risk factor for lung cancer is still subject to debate (Kheir *et al.*, 2019). While we did find EBV viral presence enriched in lung cancer (69/666, *P* = 0.000002), only two of these had detected viral integrations. In all these samples, viral sequencing depth was less than 2.5% of the host indicating that EBV is not clonally integrated into the tumour.

Integration of viral sequence into unmappable regions of the genome was dominated by herpesvirus telomeric integration as expected (Kaufer *et al.*, 2011). While recently developed optical mapping protocols are able to localise these viral integrations to specific chromosomes for inherited chromosomally integrated HHV (Wight *et al*., 2020), VIRUSBreakend can only detect the presence of telomeric integrations as it lacks the long range information required for disambiguation. The unmappable HPV integrations predominantly occurred in samples in which a mappable HPV was also found indicating that these may be passenger events. Only three HPV-16 samples contained only unmappable HPV integrations: a possibly intronic poly-GGAA; an alpha satellite integration; and a highly amplified telomeric integration. Further investigation is required to ascertain the functional relevance of centromeric and telomeric viral integrations.

### 3.4 Clinical validation

For 42 patients in the Hartwig cohort, pathology findings on viral presence were available using routine PCR testing (Supplementary Table S3) (Roepman *et al.*, 2021). Of the 38 HPV tests (QIAscreen HPV PCR Test (Qiagen)), VIRUSBreakend detected HPV integrations in the 25 HPV positive samples, and no HPV presence in the 13 HPV negative samples. VIRUSBreakend taxonomic classifications were in concordance with pathology HPV subtype determinations on the nine HPV-16, four HPV-18 and three non-16/18-HPV high risk HPV samples for which results were available. EBER immunohistochemistry (for detection of EBV protein) was performed for seven patients with VIRUSBreakend detecting EBV viral integration in the 4 EBER positive samples, and no viral integration in the three EBER negative samples. VIRUSBreakend detected the presence of EBV in one of the EBER negative samples but the coverage was less than 0.5% of the EBV positive samples. This low level of EBV is consistent with non-pathogenic historical EBV infection. Overall, VIRUSBreakend results were consistent with pathology findings for all 45 tests, including HPV high-risk subtype determination.

### 3.5 Runtime performance

To evaluate runtime performance, all callers were run on the HCC 177T sample with 4 threads specified (Fig. 3b). Each caller was allocated 4 cores and 64GB of memory on a HPC cluster containing dual socket Xeon E5-2690 servers. Both VIRUSBreakend and VERSE were using host-aligned BAM as inputs, whereas BATVI and ViFi used fastq input. VIRUSBreakend completed in 35 minutes, BATVI 41 hours, VERSE 17 hours, and ViFi 44 hours.

On the Hartwig Medical Foundation cohort, VIRUSBreakend average execution time on a 4 core 64 GB google cloud compute instance was 45 min for samples without detected virus and 85 min for samples with detected virus. VIRUSBreakend runtime is dominated by input BAM/CRAM decompression with CRAM decompression requiring more CPU usage than BAM. VIRUSBreakend supports direct streaming of input files. This eliminates the input file copy overhead when run in the cloud. Using input file streaming and preemptible c2-standard-4 instances in the Google Cloud Platform, the entire Hartwig cohort of over 500TB of CRAMs was processed for under US$500 in compute costs.

## 4 Discussion

When viruses are integrated into low mappability sequences, existing read mapped based approaches must choose between erring on the side of caution and omitting these calls, or aiming for high sensitivity at the cost of a high false discovery rate. By taking a virus-centric single breakend approach, VIRUSBreakend solves this dilemma and enables both accurate and sensitive integration detection even in regions of low mappability. This does however come at a cost. Since the single breakends must be assembled, a traditional read mapping-based caller will have greater sensitivity on shallowly sequenced samples as they do not suffer from the abrupt drop in sensitivity that VIRUSBreakend is subject to when there are not enough supporting reads for reliable assembly.

The novel single breakend approach taken by VIRUSBreakend is relevant not only to short read sequencing, but long read sequencing as well. While long reads are usually long enough to span across most simple repeats, this is not the case for the satellite repeat regions. Unless a long read viral integration detection tool uses something similar to the VIRUSBreakend single breakend approach, it will be blind to integrations in satellite repeats.

Key to the extremely fast runtime performance of VIRUSBreakend is use of host-aligned input enabling the vast majority of reads to be immediately discarded. If a host-aligned input file was not available, the computational cost of VIRUSBreakend would increase by over an order of magnitude. For the vast majority of projects, this requirement is unproblematic as a host-aligned BAM/CRAM will be created for variant calling purposes. Since VIRUSBreakend has no constraints on the host reference used (other than they not contain the viral sequences), it is suitable for immediate incorporation into an existing WGS pipeline.

Unlike existing tools, VIRUSBreakend has pan-virome integration detection capabilities and is able to detect integration sites for an arbitrary number of co-infecting viruses. This does however come with limitations. The one genome per genus ensures that a small amount of taxonomic misclassification does not result in a viral reference containing multiple related viral strains, but it also masks the presence of genuine viral co-infection by closely related viruses. Although VIRUSBreakend does report summary statistics regarding how good the within-genus taxonomic assignment is, it does not yet report co-infection by multiple viruses in the same genus.

## 5 Conclusion

The single breakend variant calling and assembly approach taken by VIRUSBreakend enables sensitive and accurate viral integration detection even in low mappability host regions. Since it combines both viral presence and integration detection into a streamlined high-speed tool, it is ideal for augmenting WGS-based sequencing pipelines with viral information and has direct clinical utility for WGS cancer patient reporting. VIRUSBreakend is a marked improvement on existing tools and provides a foundation for future research into the impact of viral integration into centromeric and telomeric regions currently considered inaccessible to short read sequencing.

## Supplementary Material

btab343_supplementary_data

## References

[btab343-B1] Brister J.R. et al (2015) NCBI viral genomes resource. Nucleic Acids Res., 43, D571–D577. 25428358 10.1093/nar/gku1207 PMC4383986

[btab343-B2] Cameron D.L. et al (2017) GRIDSS: sensitive and specific genomic rearrangement detection using positional de Bruijn graph assembly. Genome Res., 27, 2050–2060. 29097403 10.1101/gr.222109.117 PMC5741059

[btab343-B3] Cameron D.L. et al (2019) Comprehensive evaluation and characterisation of short read general-purpose structural variant calling software. Nat. Commun., 10, 3240. 31324872 10.1038/s41467-019-11146-4 PMC6642177

[btab343-B4] Cameron D.L. et al (2020) GRIDSS2: harnessing the power of phasing and single breakends in somatic structural variant detection. BioRxiv.

[btab343-B5] Chen X. et al (2019) Comprehensive comparative analysis of methods and software for identifying viral integrations. Brief. Bioinf., 20, 2088–2097. 10.1093/bib/bby070 30102374

[btab343-B6] Chen Y. et al (2013) VirusSeq: software to identify viruses and their integration sites using next-generation sequencing of human cancer tissue. Bioinformatics, 29, 266–267. 23162058 10.1093/bioinformatics/bts665 PMC3546792

[btab343-B7] Danecek P. et al; 1000 Genomes Project Analysis Group. (2011) The variant call format and VCFtools. Bioinformatics, 27, 2156–2158. 21653522 10.1093/bioinformatics/btr330 PMC3137218

[btab343-B8] Grüning B. , Bioconda Team. et al (2018) Bioconda: sustainable and comprehensive software distribution for the life sciences. Nat. Methods, 15, 475–476. 29967506 10.1038/s41592-018-0046-7 PMC11070151

[btab343-B9] Ho D.W.H. et al (2015) Virus-Clip: a fast and memory-efficient viral integration site detection tool at single-base resolution with annotation capability. Oncotarget, 6, 20959–20963. 26087185 10.18632/oncotarget.4187 PMC4673242

[btab343-B10] Huang W. et al (2012) ART: a next-generation sequencing read simulator. Bioinformatics, 28, 593–594. 22199392 10.1093/bioinformatics/btr708 PMC3278762

[btab343-B11] Jiang C. et al (2020) Distinct viral reservoirs in individuals with spontaneous control of HIV-1. Nature, 585, 261–267. 32848246 10.1038/s41586-020-2651-8 PMC7837306

[btab343-B12] Kaufer B.B. et al (2011) Herpesvirus telomeric repeats facilitate genomic integration into host telomeres and mobilization of viral DNA during reactivation. J. Exp. Med., 208, 605–615. 21383055 10.1084/jem.20101402 PMC3058580

[btab343-B13] Kheir F. et al (2019) Detection of Epstein-Barr virus infection in non-small cell lung cancer. Cancers, 11, 759. 31159203 10.3390/cancers11060759 PMC6627930

[btab343-B14] Khoury J.D. et al; TCGA Network. (2013) Landscape of DNA virus associations across human malignant cancers: analysis of 3,775 cases using RNA-Seq. J. Virol., 87, 8916–8926. 23740984 10.1128/JVI.00340-13 PMC3754044

[btab343-B15] Kosugi S. et al (2019) Comprehensive evaluation of structural variation detection algorithms for whole genome sequencing. Genome Biol., 20, 117. 31159850 10.1186/s13059-019-1720-5 PMC6547561

[btab343-B16] Li H. , Durbin R. (2010) Fast and accurate long-read alignment with Burrows–Wheeler transform. Bioinformatics, 26, 589–595. 20080505 10.1093/bioinformatics/btp698 PMC2828108

[btab343-B17] Li J.-W. et al (2013) ViralFusionSeq: accurately discover viral integration events and reconstruct fusion transcripts at single-base resolution. Bioinformatics, 29, 649–651. 23314323 10.1093/bioinformatics/btt011 PMC3582262

[btab343-B18] McLaughlin-Drubin M.E. , Munger K. (2008) Viruses associated with human cancer. Biochim. Biophys. Acta, 1782, 127–150. 18201576 10.1016/j.bbadis.2007.12.005 PMC2267909

[btab343-B19] Miga K.H. et al (2020) Telomere-to-telomere assembly of a complete human X chromosome. Nature, 585, 79–84. 32663838 10.1038/s41586-020-2547-7 PMC7484160

[btab343-B20] Nguyen N.-P.D. et al (2018) ViFi: accurate detection of viral integration and mRNA fusion reveals indiscriminate and unregulated transcription in proximal genomic regions in cervical cancer. Nucleic Acids Res., 46, 3309–3325. vol. 29579309 10.1093/nar/gky180 PMC6283451

[btab343-B21] Priestley P. et al (2019) Pan-cancer whole-genome analyses of metastatic solid tumours. Nature, 575, 210–216. 31645765 10.1038/s41586-019-1689-y PMC6872491

[btab343-B22] Roepman P. et al (2021) Clinical validation of Whole Genome Sequencing for cancer diagnostics. J Mol Diagn. 10.1016/j.jmoldx.2021.04.011 33964451

[btab343-B23] Smit A.F.A. et al (1996) RepeatMasker Open-4.0. 2013–2015.

[btab343-B24] Sung W.-K. et al (2012) Genome-wide survey of recurrent HBV integration in hepatocellular carcinoma. Nat. Genet., 44, 765–769. 22634754 10.1038/ng.2295

[btab343-B25] Tennakoon C. , Sung W.K. (2017) BATVI: fast, sensitive and accurate detection of virus integrations. BMC Bioinformatics, 18, 71. 28361674 10.1186/s12859-017-1470-x PMC5374687

[btab343-B26] Travé G. , Zanier K. (2016) HPV-mediated inactivation of tumor suppressor p53. Cell Cycle, 15, 2231–2232. 27245825 10.1080/15384101.2016.1191257 PMC5004678

[btab343-B27] Wang Q. et al (2013) VirusFinder: software for efficient and accurate detection of viruses and their integration sites in host genomes through next generation sequencing data. PLoS One, 8, e64465. 23717618 10.1371/journal.pone.0064465 PMC3663743

[btab343-B28] Wang Q. et al (2015) VERSE: a novel approach to detect virus integration in host genomes through reference genome customization. Genome Med., 7, 2. 25699093 10.1186/s13073-015-0126-6 PMC4333248

[btab343-B29] Wight D.J. et al (2020) Unbiased optical mapping of telomere-integrated endogenous human herpesvirus 6. Proc. Natl. Acad. Sci. USA. doi:10.1073/pnas.2011872117. 10.1073/pnas.2011872117 PMC7733811 33229517

[btab343-B30] Wood D.E. et al (2019) Improved metagenomic analysis with Kraken 2. Genome Biol., 20, 257. 31779668 10.1186/s13059-019-1891-0 PMC6883579

[btab343-B31] Xia Y. et al (2019) Detecting virus integration sites based on multiple related sequencing data by VirTect. BMC Med. Genomics, 12, 19. 30704462 10.1186/s12920-018-0461-8 PMC6357354

[btab343-B32] Zapatka M. et al; PCAWG Consortium. (2020) The landscape of viral associations in human cancers. Nat. Genet., 52, 320–330. 32025001 10.1038/s41588-019-0558-9 PMC8076016

