# Supplementary material for: VIRUSBreakend: Viral Integration Recognition Using Single Breakends
Source: Bioinformatics. 2021 May 11;37(19):3115–9. doi: 10.1093/bioinformatics/btab343 (PMC8504616; doi:10.1093/bioinformatics/btab343)
Supplement: btab343_supplementary_data [file btab343_supplementary_data.zip › Supplementary Methods VIRUSBreakend.docx]

Supplementary Materials

VIRUSBreakend: Viral Integration Recognition Using Single Breakends

Daniel L. Cameron, Nina Jacobs, Paul Roepman, Peter Priestley, Edwin Cuppen, Anthony T. Papenfuss

# Detailed Methods

## VIRUSBreakend pipeline

VIRUSBreakend uses a multistage approach to identifying viral integration sites. As input, it uses a SAM/BAM/CRAM file of reads aligned to the host reference genome. Sequences that do not align to the reference are classified as viral or not viral using Kraken2 2.1.1[^16^](https://paperpile.com/c/3HzASA/44dL). Viral reads are then aligned to the most abundant host-infecting virus with bwa [^30^](https://paperpile.com/c/3HzASA/TdGJ) 0.7.17, realigned to a modified viral reference that incorporates SNVs called by bcftools 1.7, single breakends identified with GRIDSS2 2.11.0[^17,18^](https://paperpile.com/c/3HzASA/78oG+MR7y), aligned to the host to identify putative integration sites, and annotated with RepeatMasker to identify false positive and multi-mapping integration sites.

All read sequences 20bp or longer that are not aligned to the host reference genome are classified using a custom Kraken2 database. For soft clipped reads, only the unaligned bases are classified. For split read alignments, only the bases not aligned to either location are classified. The full read is considered for all unmapped reads.

The custom Kraken2 database contain the standard RefSeq human, bacteria, viral and UniVec_Core sequences extracted by kraken2-build, and is augmented with all NCBI neighbour genomes genomes using *esearch -db nuccore -query "Viruses[Organism] NOT cellular organisms[ORGN] NOT wgs[PROP] NOT AC_000001:AC_999999[pacc] NOT gbdiv syn[prop] AND (srcdb_refseq[PROP] OR nuccore genome samespecies[Filter])"*. The VIRUSBreakend Kraken2 database/viral genome reference catalog can be downloaded and built using the supplied virusbreakend-build.sh script.

Sequences are considered to be of interest if either Kraken2 classifies the overall sequence with a viral taxid, any kmer is classified with a viral taxid and all kmers between that kmer are an ancestor of a viral taxid. That is, the sequence is entirely a virus of interest, or could be a split read containing a virus of interest and non-viral sequence (such as a read overlapping a host integration site).

The originating reads names for sequences of interest are tracked and a second pass over the input file is performed to extract the entire originating fragment (i.e. both reads if paired-end sequencing) for all sequences of interest.

A viral reference is created consisting of one viral genome based on the most abundant human-infecting viral taxid for each genus. Here, we define ‘most abundant’ as a viral taxid of interest with the most reads directly assigned to that taxid by Kraken2, with ties broken by selecting the taxid with the most read assigned to that taxid or any of the descendent taxid. Only taxid with at least 50 reads assigned to it or a descendant and having an associated genome sequence in the Kraken2 database are considered. If no taxid of interest reaches the 50 sequence threshold, processing terminates. When determining the viral genome to include in the viral reference, all viral genome in the database associated with the relevant taxon or any of its descendant taxa are considered. A kmer-to-genome lookup is created consisting of viral genome 16mers (and their reverse compliments) with a 16bp stride. Viral read kmers (16mers, 1bp stride, forward strand only) are compared to these genomes and the genome with the most matching input read kmers is selected for inclusion in the viral reference. All taxa with at least one associated accession included in the NCBI Viral Genomes [^31^](https://paperpile.com/c/3HzASA/h7X0) neighbours file (<https://www.ncbi.nlm.nih.gov/genomes/GenomesGroup.cgi?taxid=10239&cmd=download2>) with host containing “human” is considered human-infecting.

Extracted reads are aligned to the viral reference and SNVs called using bcftools call -c -v --ploidy 1 -V indels. The viral reference genome is then updated using bcftools consensus. To preserve viral coordinates, indels and SVs are not incorporated. Viral genomes with less than 10% of the genome covered by at least one aligned read are filtered. Reads are realigned to the updated reference and assembly/structural variant calling is performed using GRIDSS2. GRIDSS2 is invoked with `--includeIndels false --includeSR false --includeDP false` as intra-viral SVs are not required for detecting host integrations and a custom GRIDSS2 configuration file with increased assembly complexity thresholds is used reduce the chance of assembly abort in samples with very high (10,000x+) viral depth of coverage. Details can be found in the virusbreakend.sh driver scripts. To improve library fragment size distribution estimation and GRIDSS quality score calculations, GRIDSS metrics are precomputed from the first 10,000,000 reads in the host-aligned input SAM/BAM/CRAM

Candidate host genome integration positions are identified by aligning the breakend inserted sequenced to host reference genome using bwa/gridss.AnnotateInsertedSequence which annotates variants with the nominal alignment, mapq, and also any alternative alignments reported in the bwa XA tag. The VCF is annotated with gridss_annotate_vcf_repeatmasker.sh and gridss_annotate_vcf_kraken2.sh using the same custom Kraken2 database used for viral sequence identification.

The VCF is filtered to only single breakend variants in which the Kraken2 classification of the single breakend sequence matches the host NCBI taxonomy ID (human, 9606). Finally, single breakend calls are transformed into host-virus breakpoint calls based on the first alignment position reported by bwa/gridss.AnnotateInsertedSequence with the alignment mapping quality score used to determine whether the called position is ambiguous or not. Variants in which less than 50% of the breakend sequence have a host alignment are filtered. Integrations with an assembly mapping quality score less than 10 are annotated with a LOW_MAPQ in the VCF FILTER column. A tsv is also produced containing summary details about the detected viruses and integrations.

## Tools

VIRUSBreakend 2.11.0, BATVI 1.03, VERSE 2.0, ViFi (commit d56f4c2) and GRIDSS 2.11.0 were run with default settings using the installation procedures outlined in their user guide. All VERSE perl scripts were edit to use “#!/bin/usr/env perl” so as to be compatible with a conda installation, the base qual cutoff for the embedded CREST was reduced to 15 since wgsim defaults to 17 as a base quality score, and the undocumented perl dependencies were iteratively installed until execution no longer raised missing library errors. The missing step of creating bwa index in the BATVI batmis directory was run in addition to the installation instructions to circumvent the fatal error encountered building the indexes when following the documented instructions. BATVI full call set results (predictions.opt.subopt.txt) were not included as the recall of this call set was lower than the BATVI high confidence calls on the simulation data. The following modifications were required to get the most recent version (27 Mar 2019 d56f4c28) of ViFi to run: recommendation to use the supplied docker image was ignored since the docker command-line parsing logic was incorrect and crashed when using -b, and ignored the -v parameter thus always ran against HPV; crash bug in scripts/get_trans_new.py:238 was fixed by correcting the incorrect parenthesis on line 233; a conda environment was created using “conda create -n vifi bwa=0.7.17 python=2.7 pysam=0.15.2 samtools=1.9 hmmer”. VIRUSBreakend was run using GRIDSS version 2.11.0, 4 threads and --rmargs "-e rmblast" since the conda installation of RepeatMasker does not perform RepeatMasker configuration. RepeatMasker 4.1.0 and Kraken 2.1.1 were installed from BioConda [^32^](https://paperpile.com/c/3HzASA/xrhZ). Reads were aligned to hg19 using bwa mem 0.7 and converted to bam and coordinate sorted using samtools 1.11.

## Synthetic benchmark

Viral integration sites were simulated by generating a fasta consisting of the 50kbp of host sequence before the insertion side, 2,000bp of HBV viral sequence, a 10bp host gap, then the 50kbp of host sequence after the insertion site. To enable accurate simulation of integration in telomeric and centromeric repeats, chromosome 1 of the CHM13 Telomere-to-Telomere consortium assembly (<https://github.com/nanopore-wgs-consortium/CHM13>) was used as the host sequence. The non-reference strain LC500247.1 was used as the viral sequence.

248 integration sites were simulated, one at each 1Mb position along CHM13 chr1 (position chr1:1000000, chr1:2000000, ...), each with a different HBV integration position. For determining repeat performance , 2,483 integration sites were simulated, each 100kbp after the previous (chr1:100,000, chr1:200,000, …). Simulated reads were generated using ART 2.5.8[^21^](https://paperpile.com/c/3HzASA/HSq8) with parameters --noALN --paired --seqSys HSXn -ir 0 -ir2 0 -dr 0 -dr2 0 -k 0 -l 150 -m 500 -s 100 -rs 1. Separate data sets were generated with coverage (--fcov) of 5, 10, 15, 30, and 60. Each was called with VIRUSBreakend, VERSE, ViFi, BATVI, and GRIDSS2 using their default hg19 settings. GRIDSS2 was included due to its favorable performance as a general purpose breakpoint caller[^22,23^](https://paperpile.com/c/3HzASA/Gdwd+GX97) as well as to evaluate the performance of host-centric single breakend calling. True positives required host coordinates to match within 1000bp (or 1Mbp for locations without a 1-to-1 CHM13 to hg19 coordinate mapping) and viral coordinates to within 750bp (to account for different HBV viral references). To ensure VIRUSBreakend was not favoured by its inclusion of LC500247 in its viral database, its viral database was restricted to RefSeq sequences only. GRIDSS metrics from 20M simulating reads from chm13 using the same parameters were used to emulate realistic VIRUSBreakend WGS metrics calculations as no simulation data set contained the 10M reads used for metrics approximation. An E. Coli read pair and an E Coli/human read pair was appended to each fastq file to ensure ViFi did not crash. VIRUSBreakend was run with --minreads 15 to ensure the viral presence filtering did to interfere with this benchmark of integration detection capability. GRIDSS2 single breakend calls were filtered to variants that realigned to HBV using gridss.AnnotateInsertedSequence.

To account for coordinate differences between hg19 and CHM13 and LC500247.1 and the HBV references used by the callers, calls were considered a true positive if the hg19 host position and CHM13 truth positions matched, and the viral position was within 750bp of the LC500247.1.

hg19/CHM13 positions were considered matching if CHM13 position lifted over (<http://t2t.gi.ucsc.edu/chm13/hub/t2t-chm13-v1.0/hg38Lastz/t2t-chm13-v1.0.hg38.over.chain.gz>) to hg38 lifted over (R liftOver package) to hg19 were within 1kb, or within 1Mb in the case of lifftOver failure. Calls were considered homologous calls if the viral position matched and no full matches were found for that breakpoint. Duplicate or unmatched calls were considered false positives. GRIDSS2 breakpoint calls were generated by concatenating NC_003977.2 to hg19 aligning using bwa mem 0.7.17 and filtering to breakpoint calls involving NC_003977.2.

CHM13 RepeatMasker annotations were downloaded from (<https://s3.amazonaws.com/nanopore-human-wgs/chm13/assemblies/annotation/chm13.draft_v1.0_plus38Y_repeatmasker.out.gz>) and insertion sites annotated with the at the insertion position. Homologous calls were considered true positives.

## Hepatocellular Carcinoma Benchmark

Reads associated with samples 145T, 177T, 180N, 186T, 198T, 26T, 200T, 268T, 43T, 46T, 70T, 71T, 95T in project ERP001196 were downloaded from SRA using fasterq-dump from sra-tools 2.10.8. ERR093473 and ERR173541 were excluded from analysis due non-transient fasterq-dump errors that could not be rectified. Since VIRUSBreakend supports multiple input files and GRIDSS performs per-file library fragment size distribution estimation, bam files were not merged. GRIDSS calls were annotated with gridss_annotate_kraken2.sh and filtered to single breakends with a viral taxid. VERSE and ViFi were not rerun and the results presented in their respective publications were taken as is.

## Metastatic solid tumour cohort

VIRUSBreakend was run on 5,191 samples in the Hartwig Medical Foundation cohort using Google Cloud Engine pre-emptible 4 vCPUs, 64 GB memory instances. TTV and xenotropic retroviruses were excluded from counts.

# Availability of data and materials

VIRUSBreakend is available as free and open source software under a GPLv3 license and is available at <https://github.com/PapenfussLab/VIRUSBreakend/>

Benchmarking and figure generation scripts can be found at <https://github.com/PapenfussLab/gridss/tree/master/scripts/virusbreakend_manuscript>

HCC cohort data was downloaded from ERA project ERP001196.

Hartwig Medical Foundation cohort data was obtained from the Hartwig Medical Foundation (Data request DR-005). Standardized procedures and request forms for access to this data can be found at<https://www.hartwigmedicalfoundation.nl/en>

# Author contributions

DLC designed and implemented VIRUSBreakend. DLC performed experiments. NJ, PR provided clinical validation and interpretation, PP, EC, ATP performed critical assessments of results and tools. DLC, EC, ATP contributed to writing of the manuscript. All authors read and approved the manuscript.
